# High-resolution structural analysis of the cyanobacterial photosystem I complex reveals independent incorporation of small transmembrane and cytoplasmic subunits

**DOI:** 10.1016/j.xplc.2025.101493

**Published:** 2025-08-25

**Authors:** Quentin Charras Ferroussier, Sadanand Gupta, Martin Tichý, Ashraf Al-Amoudi, Martin Lukeš, Daniel Štipl, Peter Koník, David Bína, Marek Zákopčaník, Petr Novák, Roman Sobotka, Josef Komenda, Andreas Naschberger

**Affiliations:** 1 King Abdullah University of Science and Technology (KAUST), Biological and Environmental Science and Engineering Division, 23955 Thuwal, Saudi Arabia; 2 Centre Algatech, Institute of Microbiology of the Czech Academy of Sciences, 379 01 Třeboň, Czech Republic; 3 Faculty of Science, University of South Bohemia, 370 05 České Budějovice, Czech Republic; 4 Laboratory of Structural Biology and Cell Signaling, Institute of Microbiology of the Czech Academy of Sciences, 14220 Prague, Czech Republic; 5 Institute of Plant Molecular Biology, Biology Centre of the Czech Academy of Sciences, 370 05 České Budějovice, Czech Republic

Dear Editor,

Photosynthetic energy conversion, essential for life on Earth, is performed by cyanobacteria, algae, and plants using photosystem I (PSI) and II (PSII). These membrane complexes consist of numerous protein subunits and cofactors, including chlorophylls (Chls), carotenoids, quinones, and lipids. They transfer electrons from water to NADP^+,^ which is then used for CO_2_ fixation together with ATP. The PSI core consists of the large transmembrane (TM) subunits PsaA and PsaB (Caspy et al., 2020), which hold cofactors required for electron transfer, including special Chls, quinones, and the [4Fe–4S] cluster F_X_. Cytoplasmic PsaC, stabilized by its neighboring PsaD and PsaE subunits, binds two additional [4Fe–4S] clusters, F_A_ and F_B_, which accept electrons from F_X_ and reduce ferredoxin or flavodoxin, driving downstream metabolic processes (Caspy et al., 2020; Kanda et al., 2021). Smaller TM subunits, including PsaF, PsaI, PsaJ, PsaK, PsaL, and PsaM, are also important for the optimal function and structure of PSI (Malavath et al., 2018; Caspy et al., 2020). PSI typically forms trimers in cyanobacteria (Malavath et al., 2018) but exists as monomers or dimers in algae (Naschberger et al., 2022) and plants (Caspy et al., 2020). Although the structure of mature PSI has been extensively characterized, its assembly in photosynthetic cells remains to be fully clarified. PSI assembly steps are typically seen as sequential, but the exact order in which small subunits and auxiliary assembly factors are added after formation of the PsaA/PsaB heterodimer (Wostrikoff et al., 2004) is still largely unknown.

Previous studies have proposed that binding of the stromal/cytoplasmic subunits PsaC, PsaD, and PsaE is preceded by coordination of the F_X_ iron–sulfur cluster (Kanda et al., 2021) into the early-formed PsaA/PsaB heterodimer and is followed, notably in plants, by incorporation of the small TM subunits PsaL, PsaI, and PsaH (Rolo et al., 2024). In cyanobacteria, it has been proposed that PsaK is incorporated at the latest stage of PSI monomer assembly (Dühring et al., 2007) and that the presence of PsaM (Naithani et al., 2000) and especially PsaL (Dühring et al., 2007) is required for formation of the stable PSI trimer (Figure 1A). However, this model is challenged by the observation that the small TM subunits can still be incorporated in the absence of cytoplasmic subunits in a rubredoxin A knockout mutant of *Synechococcus (*Shen et al., 2002). To provide new insights into PSI biogenesis, we analyzed the structure of a monomeric PSI complex isolated from the ΔPsaC mutant of the cyanobacterium *Synechocystis sp.* PCC 6803 (hereafter *Synechocystis*). Using single-particle cryoelectron microscopy (cryo-EM), its structure was resolved at an exceptionally high resolution of 1.83 Å, providing new insight into PSI assembly.

**Figure 1:**
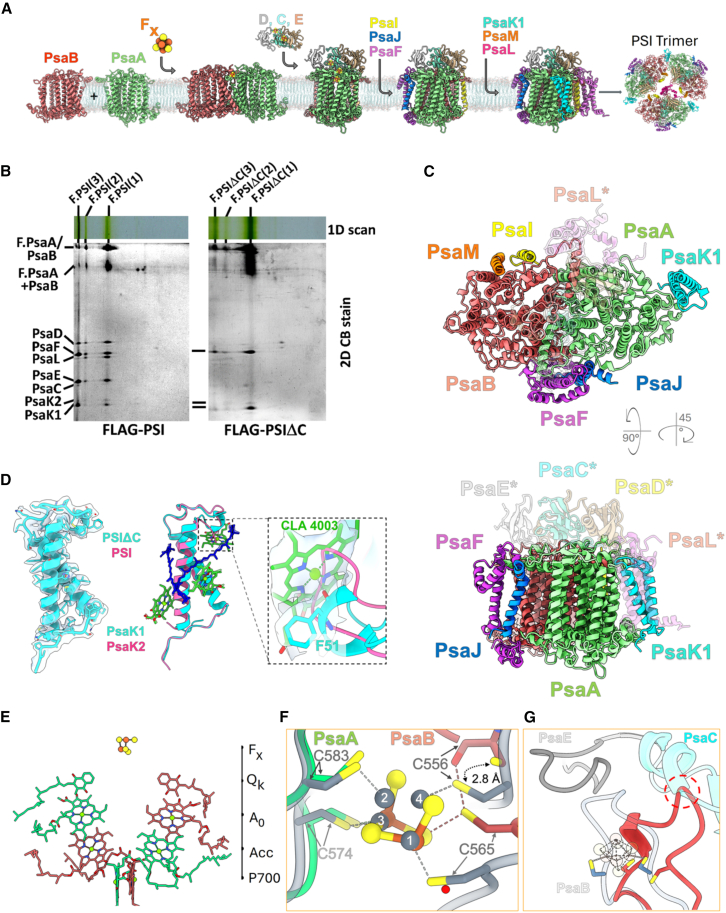
New insights into PSI assembly in cyanobacteria. **(A)** Current model of PSI assembly. **(B)** CN–PAGE of FLAG-affinity-purified pull-downs isolated from *Synechocystis* strains expressing FLAG–PsaA instead of PsaA in the presence (FLAG–PSI) or absence (FLAG–PSIΔC) of PsaC. The first-dimension native gel was photographed (1D color). After SDS–PAGE in the second dimension, the gel was stained with Coomassie Brilliant Blue (CBB). Complexes: F.PSI(3), F.PSI(2), and F.PSI(1), FLAG-tagged trimeric, dimeric, and monomeric PSI; F.PSIΔC(3), F.PSIΔC(2), and F.PSIΔC(1), FLAG-tagged trimeric, dimeric, and monomeric PSI lacking PsaC. F.PsaA/PsaB is a heterodimer of FLAG–PsaA and PsaB. Each loaded sample contained 5 μg of Chl. The structure corresponding to the F.PSIΔC(1) monomer band was resolved in the present study. **(C)** The structure of PSIΔC obtained using cryo-EM. Cytoplasmic (top) and side (bottom) views of the superposition of the PSIΔC model onto the structure of mature PSI; specific subunits of mature PSI (PDB: 5OY0) that are missing in PSIΔC are shown in transparency and designated by asterisks. Only the protein backbone is shown in cartoon representation. **(D)** Detailed analysis of the PsaK subunit in PSIΔC compared with mature PSI. Left: model and density of PsaK1 apoprotein from PSIΔC. Middle: superposition of PsaK1 and PsaK2 holoproteins from PSIΔC and mature PSI, respectively. Right: close-up view of the Chl-*a* 4003 region of PSIΔC. The models of PSIΔC (cyan) and PSI (pink) were superposed, and the density from PSIΔC is shown. **(E)** Components of the electron transport chain in PSIΔC. The cofactors are colored to indicate their association with PsaA (green) or PsaB (red). Chls of the electron transport chain are arranged in three pairs referred to as P700, Acc, and A0. Phylloquinones are labeled as Q_k_ and the iron-sulphur cluster as F_X_. **(F)** Superposition of PSIΔC (green and red) and mature PSI (gray), showing F_X_ cluster coordination by cysteine residues. Only the iron atoms and their numbers in F_X_ of mature PSI are shown in gray. **(G)** Wide view of the F_X_ coordination site. Models of PSIΔC and PSI are superposed. The PsaB loop (light gray), PsaE (gray), PsaC (cyan), and the F_X_ cluster (transparent) from mature PSI and the PsaB loop (red) from PSIΔC are shown. For both models, the Cys556 and Cys565 residues are shown. The red circle indicates the virtual clash between PsaB from PSIΔC and PsaC from the mature PSI.

We generated a *Synechocystis* mutant lacking the *psaC* gene and expressing FLAG-tagged PsaA (FLAG–*psaA*/Δ*psaC*; Supplemental Figure 1). Clear native polyacrylamide gel electrophoresis (CN–PAGE) indicated a prevalence of monomeric PSI in membranes of the mutant, with some trimeric form present, whereas the trimeric form predominated over the monomeric form in the control line (FLAG–*psaA*) (Figure 1B; Supplemental Figure 2). This result showed that depletion of PsaC substantially impairs PSI trimerization. Using FLAG affinity pull-down, we isolated FLAG–PSIΔC and control FLAG–PSI preparations. Their absorption spectra showed similar overall pigment contents (Supplemental Figure 3A), and their light–dark absorption difference spectra also demonstrated similar primary charge-separation activity (Supplemental Figure 3B). Two-dimensional CN/SDS–PAGE and mass spectrometry (MS) confirmed the presence of PsaC, PsaD, and PsaE in FLAG–PSI but their complete absence in FLAG–PSIΔC (Figure 1B; Supplemental Tables 1 and 2). By contrast, PsaF and PsaK1 were present in monomers and trimers of both preparations (Supplemental Table 3). PsaL was identified by MS in trimeric PSI from both preparations and in the FLAG–PSI monomer but only in a small fraction of the FLAG–PSIΔC monomer (Supplemental Table 3). This finding was confirmed by 2D blot analysis (Supplemental Figure 4). Bands corresponding to PsaI, PsaJ, and PsaM were not detected because of their small size and poor gel resolution (Figure 1B). Moreover, these mini-subunits yield no or a very limited number of mostly hydrophobic and barely detectable tryptic peptides, which also explains their absence in the analysis of individual native PSI bands from CN gels (Supplemental Table 3).

To determine the three-dimensional (3D) structure of the PSI complex lacking PsaC and to identify all associated subunits, the FLAG–PSIΔC preparation was analyzed by single-particle cryo-EM. For the reference and template model, we used the *Synechocystis* trimeric PSI complex resolved by Malavath et al. (2018) (PDB: 5OY0; referred to as mature PSI). We collected 9991 movies (Supplemental Figure 5, Supplemental Table 4), which enabled us to obtain 334 941 particles used to reconstruct the structure. The well-defined density enabled us to build an atomic model of the monomeric FLAG–PSIΔC complex (for simplicity, PSIΔC hereafter) with a global resolution of 1.83 Å, although the dimeric and trimeric FLAG–PSIΔC complexes were not resolved, likely because of their low abundance in the preparation. The density in the center of PSIΔC revealed high-resolution details, enabling the clear identification of pigments and their puckering state, correct rotameric angles of protein side chains, and precise conformations and orientations of Chl molecules (Supplemental Figures 6 and 7). The complex consisted of seven protein subunits: PsaA, PsaB, PsaF, and PsaK, together with PsaI, PsaJ, and PsaM, which were not detected in the proteomic analysis (Figure 1C). We clearly identified the PsaK1 paralog in PSIΔC, whereas the mature PSI reference model (PDB: 5OY0) contained PsaK2 (Figure 1D). However, the lower resolution reported in (Malavath et al. (2018) may have limited the ability to clearly identify the correct paralog, leaving open the possibility that PDB: 5OY0 also contained PsaK1. In agreement with Toporik et al. (2019), our data support predominant incorporation of PsaK1, which stabilizes Chl 4003 with an α helix through the F51 residue. By contrast, PsaK2 has a loop in this region, which would cause structural clashes in the mature PSI complex (Figure 1D). Furthermore, the absence of PsaD, which is the main interactor of PsaL at the cytoplasmic side of PSI, with up to seven contact points (Supplemental Figure 8), renders the interaction of PsaL with monomeric PSIΔC less stable than that with the complete PSI monomer (Supplemental Figure 8). This likely explains the absence of PsaL in the PSIΔC structure.

We modeled 93 Chls and 20 carotenoids in our PSIΔC structure (Supplemental Figure 7; Supplemental Table 5), along with reaction center components like P700, auxiliary Chls, and phylloquinones, organized identically to those in mature PSI (Figure 1E). In PSIΔC, the F_X_ cluster had weaker density, likely due to partial occupancy, and contained only two iron ions and four sulfide ions (Supplemental Figure 9A). In mature PSI, the F_X_ cluster comprises four sulfide anions and four ferrous cations coordinated by four cysteine residues: Cys574 (Fe3) and Cys583 (Fe2) of PsaA, and Cys556 (Fe4) and Cys565 (Fe1) of PsaB (Figure 1F; Supplemental Figure 9B). In PSIΔC, Fe1 is present but is not coordinated by its usual Cys565 residue from PsaB, which is displaced and replaced by a water molecule (Figure 1F). Instead, Cys565 interacts with the carbonyl of Cys556, and its thiol group interacts with the S2 sulfide (Figure 1F; Supplemental Figure 9C). Fe2 is absent, although its coordinating cysteine from PsaA is correctly positioned (Figure 1F). Fe3 is present and properly coordinated, similar to that in the mature complex, but Fe4 is absent, with its coordinating cysteine from PsaB displaced by 2.8 Å (Figure 1F). In the absence of PsaC, the observed loss of two irons may be the result of oxidation or another modification that occurred during purification of the complex and/or its embedding into the EM grid. Nevertheless, we believe that it is a consequence of the unavailability of Cys residues of PsaB, related to the disorder of the neighboring PsaB region (residues 573–585) (Figure 1G). This region normally includes a β-turn (577–581) stabilized by interactions with PsaC and PsaE (Figure 1G; Supplemental Figure 9D), which ensures the correct positioning of Cys556 and Cys565 of PsaB to fully coordinate the complete F_X_ cluster. This interpretation is supported by the observation of incomplete [3Fe–4S] clusters in cells of a C565S PsaC *Synechocystis* mutant and a similar *Synechococcus* mutant (Warren et al., 1993; Pérez et al., 2018). Conversely, lack of F_X_ has been shown to prevent assembly of the cytoplasmic subunits (Shen et al., 2002). Together, our data and previous studies thus suggest an intrinsically encoded mechanism for F_X_ insertion and stabilization, working through synchronized structural rearrangement of PsaB, which accompanies the binding of the cytoplasmic subunits.

In conclusion, we found that the small TM subunits of cyanobacterial PSI can associate with the PsaA/PsaB heterodimer during the assembly process, independently of the presence of cytoplasmic subunits. PsaC is essential for binding of the other two cytoplasmic subunits and also contributes to recruiting PsaL, which strongly interacts with PsaD and also facilitates trimer formation. PsaC is also important for formation of the correct F_X_ binding site, which requires conformational rearrangements of PsaB induced by PsaC binding. Without these rearrangements, the resulting F_X_ cluster remains incomplete and lacks two iron ions. Our findings allow for alternative scenarios of PSI assembly (alongside the prevailing model shown in Figure 1A), in which the simultaneous building of the F_X_ cluster and the association of PsaC (and other cytoplasmic subunits) with the PsaA/PsaB heterodimer occur after, or concurrently with, the binding of most of the small TM subunits.

## Data availability

Atomic coordinates of PSIΔC have been deposited in the Protein Data Bank under accession code 8ZWB. The EM density map of PSIΔC has been deposited in the Electron Microscopy Data Bank under accession code EMD-60522.

## Funding

We are very grateful to the 10.13039/501100004052KAUST Opportunity Fund (A.N., J.K., and R.S., project RFS-OFP2023-5561), the Czech Grant Agency (J.K.; project no. 24-10227S), ERC (J.K.; project no. 854126), the Ministry of Education, Youth and Sports (R.S. and P.N.; OP JAK project no. CZ.02.01.01/00/22_008/0004624), and Czech Academy of Sciences (D.B.; institutional support RVO:60077344) for funding.

## Acknowledgments

We thank Jan Pilný, Eva Prachová, and Kateřina Novotná for excellent technical support. We thank the cryo-EM facility at KAUST for their assistance with data collection. The authors declare no competing interests.

## Author contributions

A.N. and J.K. designed the study; S.G. and M.T. constructed the strains; S.G., R.S., D.S., and J.K. prepared and characterized the PSI complexes; D.B. recorded and analyzed the light–dark absorption difference spectra; A.N. processed the structural data; Q.C. built the model and analyzed the structure; A.A.-A. collected the cryo-EM data; M.L. and P.K. analyzed protein bands in the gel; P.N. and M.Z. performed MS analysis of the FLAG–PSIΔC preparation; and Q.C., S.G., A.N., J.K., and R.S. jointly prepared the figures and wrote the manuscript.

## Supplemental information

Document S1. Supplemental Figures 1–9, Supplemental Tables 1–6, and supplemental methods

Document S2. Article plus supplemental information
