# Supplementary material for: High-resolution structural analysis of the cyanobacterial photosystem I complex reveals independent incorporation of small transmembrane and cytoplasmic subunits
Source: Plant Commun. 2025 Aug 25;6(12):101493. doi: 10.1016/j.xplc.2025.101493 (PMC12744741; doi:10.1016/j.xplc.2025.101493)
Supplement: Document S1. Supplemental Figures 1–9, Supplemental Tables 1–6, and supplemental methods [file mmc1.pdf]

Plant Communications, Volume *6*

Supplemental information

High-resolution structural analysis of the cyanobacterial photosystem I complex reveals independent incorporation of small transmembrane and cytoplasmic subunits

Quentin Charras Ferroussier, Sadanand Gupta, Martin Tichý, Ashraf Al-Amoudi, Martin Lukeš, Daniel Štipl, Peter Koník, David Bína, Marek Zákopčaník, Petr Novák, Roman Sobotka, Josef Komenda, and Andreas Naschberger

Suppplemental Information:

Supplemental Figure 1. Construction of the *Synechocystis* mutants.

Supplemental Figure 2. Analysis of membranes from *Synechocystis* strains expressing the FLAG–PsaA subunit instead of PsaA and either containing PsaC (*FLAG–psaA*) or lacking PsaC (*FLAG–psaA/∆psaC*).

Supplemental Figure 3. Characterization of the isolated FLAG−PSI and FLAG−PSIΔC preparations.

Supplemental Figure 4. 2D Western blot analysis of FLAG–affinity purified pull-downs isolated from *Synechocystis* strains expressing Flag–PsaA instead of PsaA and either containing (FLAG–PSI) or lacking PsaC (FLAG–PSI∆C).

Supplemental Figure 5. (A) Cryo–EM data processing, (B) local resolution, and (C) angular distribution, and Fourier shell correlation plot.

Supplemental Figure 6. High resolution features of pigments and protein parts in the protein core of the PSIΔC.

Supplemental Figure 7. Pigment binding sites in the PSIΔC and HPLC analysis of pigments extracted from the isolated PSI preparations.

Supplemental Figure 8. Main protein–protein interactions established by PsaL with other subunits of mature PSI.

Supplemental Figure 9: Organization of the F_X cluster in PSIΔC and mature PSI (5OY0).

Supplemental Table1. Mass spectrometric identification of protein bands designated in the 2D gel analysis of the FLAG–PSI preparation in Fig. 1B.

Supplemental Table 2. List of the most abundant 40 proteins identified by MS in the FLAG–PSI∆C preparation.

Supplemental Table 3. Mass spectrometric (MS) and Western blot (WB) identification of proteins in trimeric and monomeric PSI complexes of FLAG–PSI and FLAG–PSI∆C preparations separated in the CN gel shown in Supplemental Figure 4.

Supplemental Table 4. Cryo–EM single particle analysis. Summary of the data collection, refinement, and validation statistics of PSIΔC (EMDB-60522) (PDB 8ZWB).

Supplemental Table 5. Pigment and lipids composition of each PSIΔC subunit based on the cryo–EM structure.

Supplemental Table 6. List of primers used for constructing mutants used in the study (see Supplemental Figure 1).

Methods

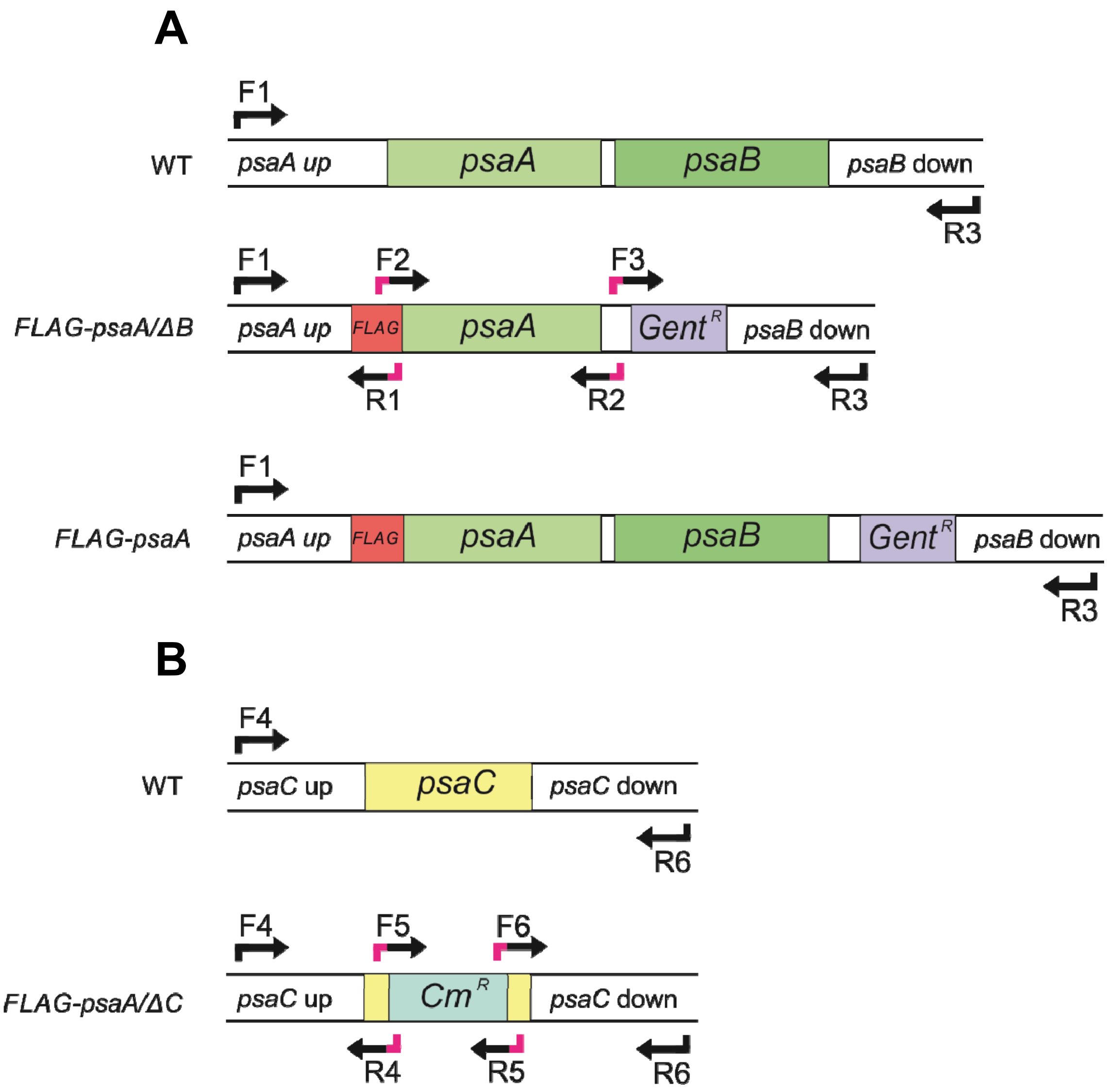

Supplemental Figure 1. Construction of the *Synechocystis* mutants. (a) The *FLAG–psaA*/Δ*psaB* strain was prepared by replacement of the original *psaA* and *psaB* genes by a construct containing *psaA* gene with an N-terminally attached sequence encoding 1xFLAG and gentamycin resistance cassette downstream. The *FLAG–psaA* strain was prepared by the insertion of the original *psaB* gene between *FLAG–psaA* gene and gentamycin resistance cassette and selecting for autotrophy. (b) The *FLAG–psaA*/Δ*psaC* strain was prepared by replacing most of the original *psaC* gene by the chloramphenicol resistance cassette. F1-F6 and R1-R6 designate primers described in Supplemental Table 6.

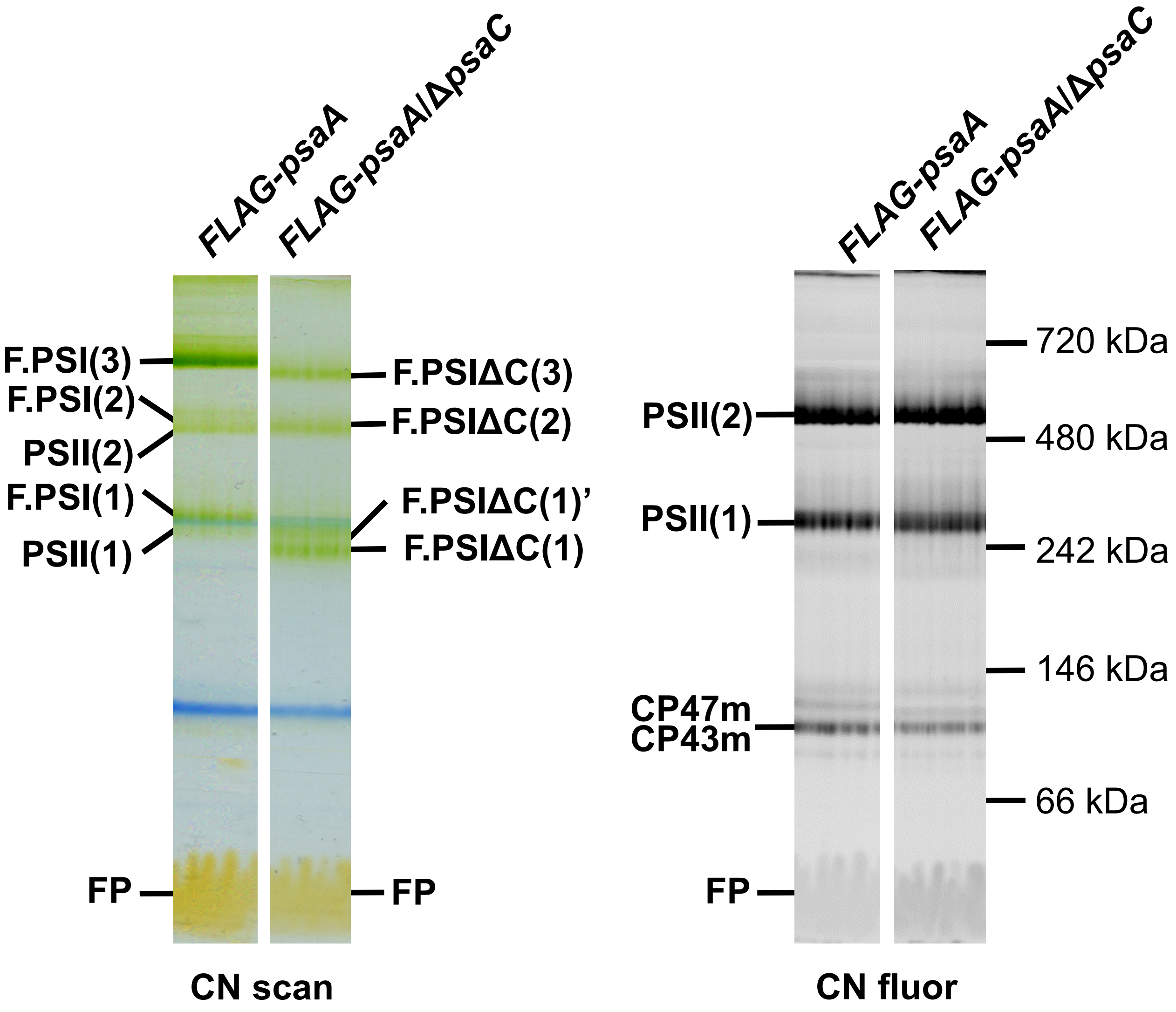

Supplemental Figure 2. Analysis of membranes from *Synechocystis* strains expressing the FLAG–PsaA subunit instead of PsaA and either containing PsaC (*FLAG–psaA*) or lacking PsaC (*FLAG–psaA/ΔpsaC*). The complexes were separated by CN PAGE and the gel was photographed (CN scan) and scanned for Chl fluorescence (CN fluor). Note the different PSI and identical PSII mobilities of complexes in the control and in the PsaC–lacking strain. Designation of complexes: F.PSI(3), F.PSI(2) and F.PSI(1), FLAG-tagged trimeric, dimeric and monomeric Photosystem I; F.PSI(3)ΔC and F.PSI(2)ΔC, FLAG-tagged trimeric and dimeric Photosystem I lacking PsaC; F.PSI(1)ΔC' and F.PSI(1)ΔC, FLAG-tagged monomeric Photosystem I lacking PsaC and containing PsaL, respectively; PSII(2) and PSII(1), dimeric and monomeric Photosystem II; CP47m and CP43m, unassembled CP47 and CP43 modules; FP, free pigments. The loading of the membranes was based on the same OD_{750nm} corresponding to 5 μg of chlorophyll for *FLAG–psaA* strain and 4 μg of chlorophyll for *FLAG–psaA/ΔpsaC* strain.

A

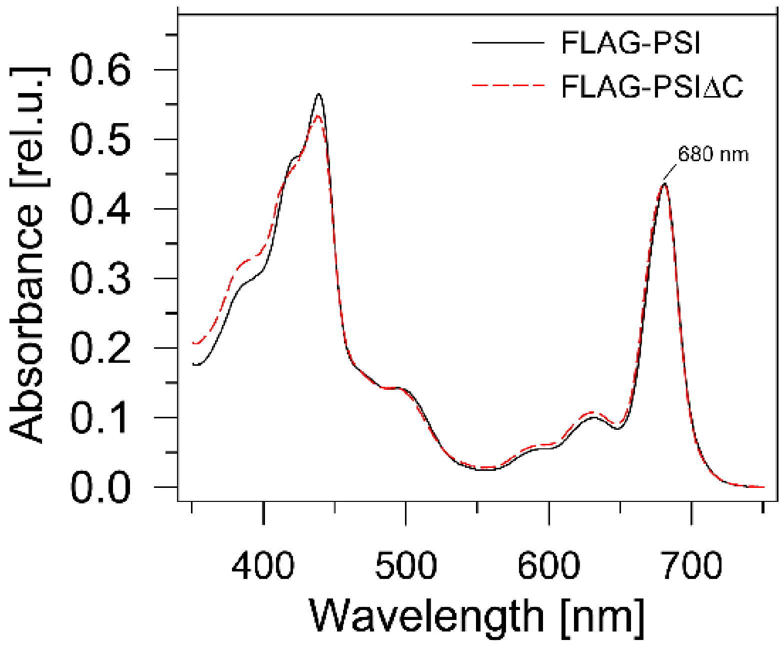

B

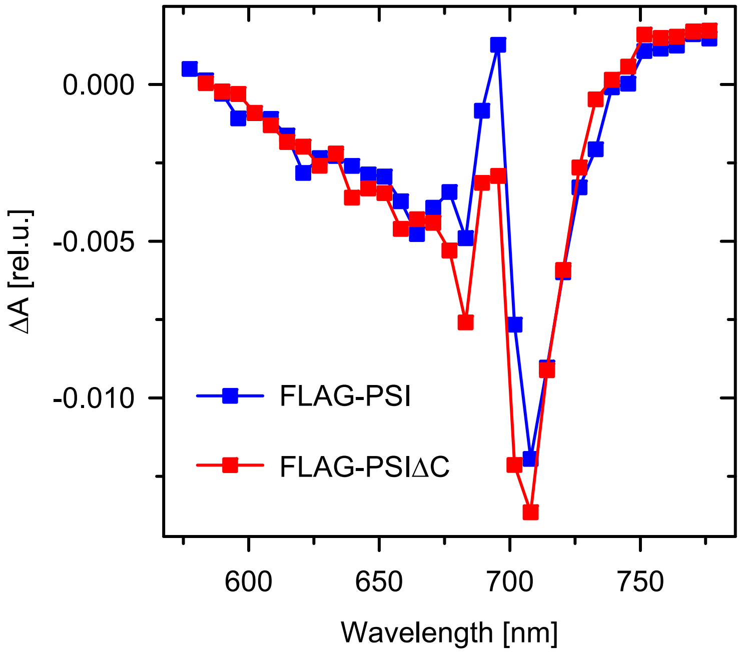

Supplemental Figure 3. Characterization of the isolated FLAG–PSI and FLAG–PSIΔC preparations. (A) Absorption spectra of FLAG-affinity purified pull-downs normalized to the 680 nm peak of Chl. **(B)** Difference (light-dark) absorption spectra measured at the end of the 3 s-long illumination of the FLAG-affinity purified pull-downs using blue LED (maximum 470 nm) in the presence of 5 mM sodium ascorbate. The spectra are normalized to the 680 nm peak of Chl.

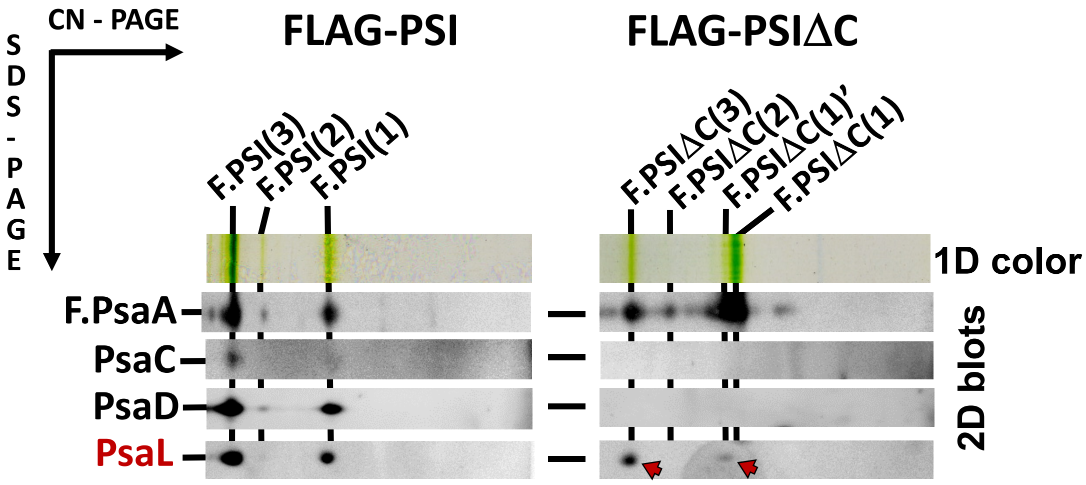

Supplemental Figure 4. 2D Western blot analysis of FLAG–affinity purified pull-downs isolated from *Synechocystis* strains expressing Flag-PsaA instead of PsaA and either containing (FLAG–PSI) or lacking PsaC (FLAG–PSIΔC). Preparations were analyzed by CN PAGE in the first dimension, the native gel was photographed (1D color), the lanes were cut and heated in 2% SDS at 50 °C for 20 min. After SDS–PAGE in the 2nd dimension the gel was stained with SYPRO Orange, electroblotted and the blot was sequentially probed by antibodies specific for PsaL, PsaC, PsaD and FLAG. Designation of complexes as in Fig. 1. The signals of PsaL in the trimer and minor monomer of FLAG–PSIΔC are designated by red arrow. Each loaded sample contained 5 μg of Chl.

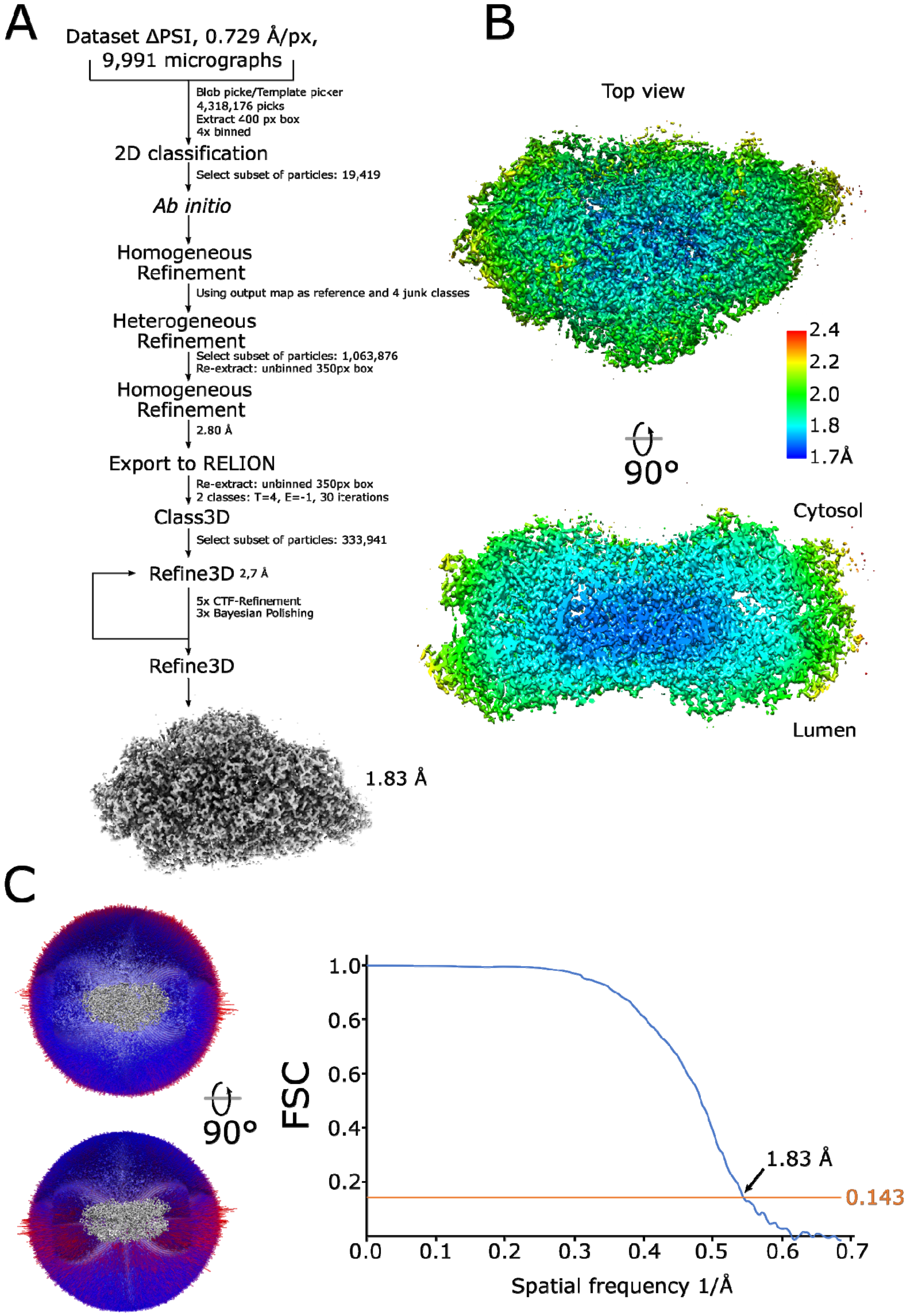

Supplemental Figure 5. (A) Cryo EM data processing, (B) local resolution, and (C) angular distribution, and Fourier shell correlation plot.

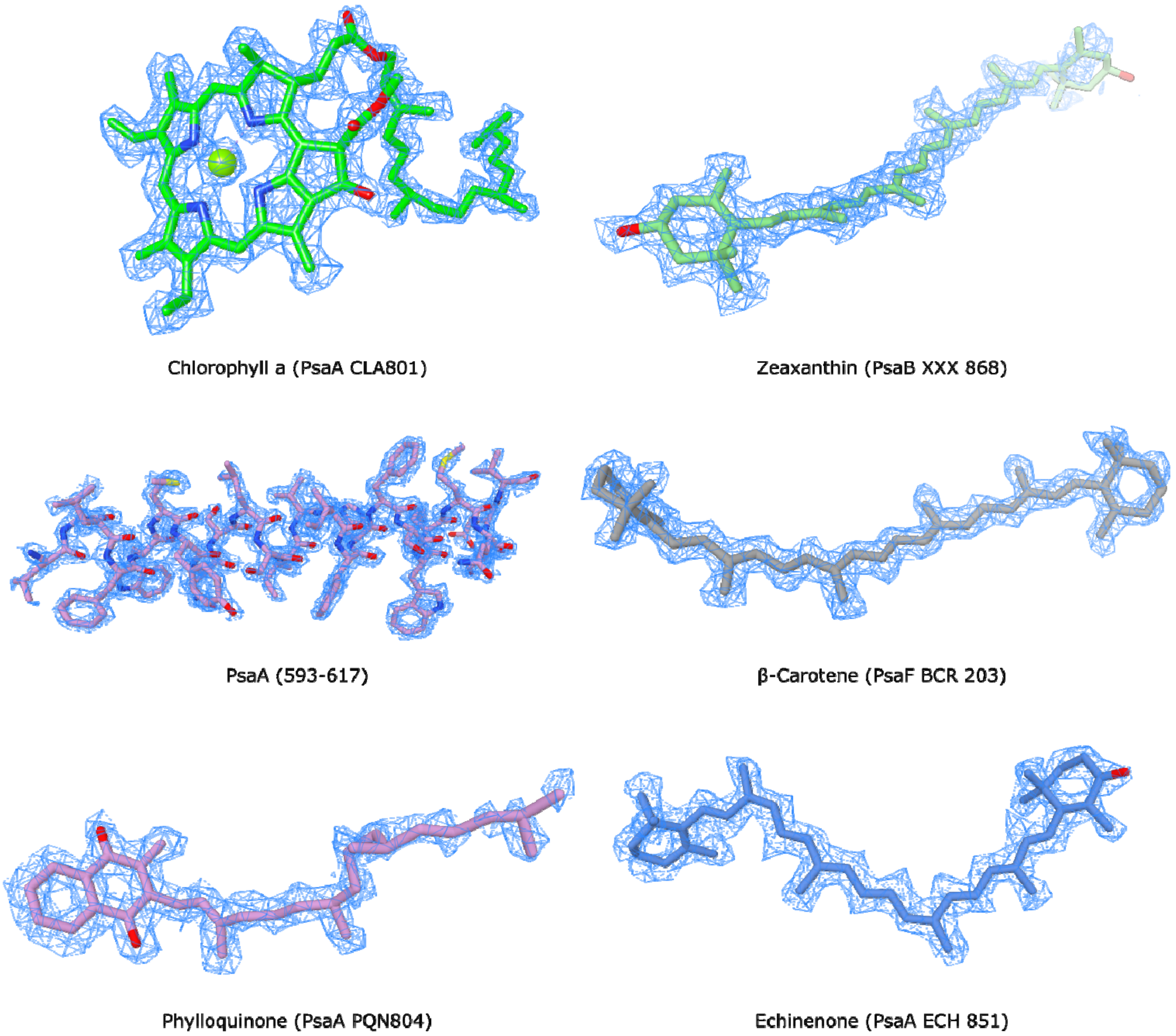

Supplemental Figure 6. High resolution features of pigments and protein parts in the protein core of the PSIΔC.

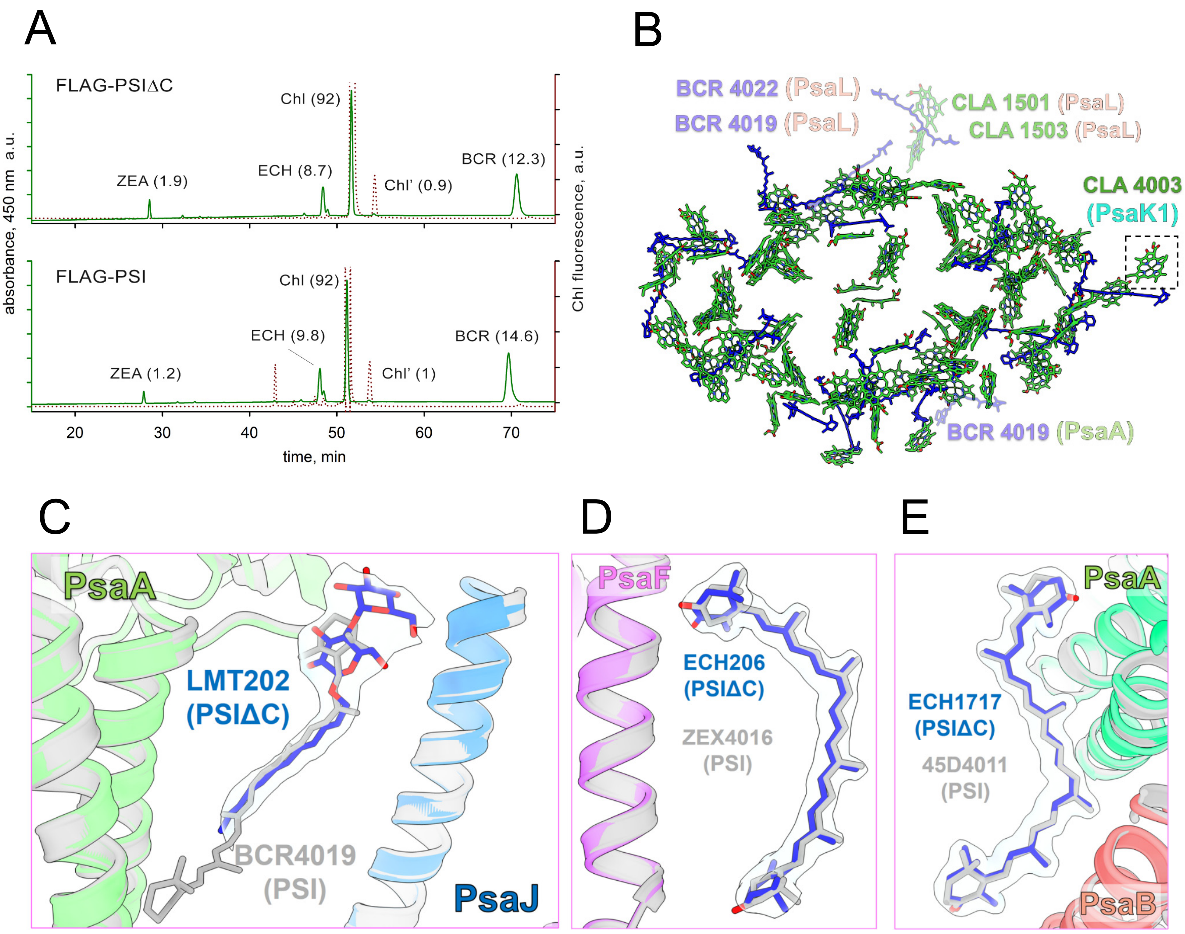

Supplemental Figure 7. Pigment binding sites in the PSIΔC and HPLC analysis of pigments extracted from the isolated PSI preparations. (A) HPLC analysis of pigments. FLAG–PSIΔC and the control FLAG–PSI pulldowns that were isolated using FLAG affinity chromatography from strains expressing FLAG–PsaA instead of PsaA and containing or lacking PsaC. The extracted carotenoids and Chl were separated by HPLC and detected by a diode-array detector (DAD) at 450 nm (solid green light). Although the $C13^2$-epimer of chlorophyll *a* (Chl') can be detected using absorbance, its quantification is not precise due to its too weak signal, specifically when compared to the absorbance of Chl. However, the fluorescence emission of the Chl' epimer at 670 nm can be detected using more sensitive fluorescence detector after excitation at 435 nm (dotted red line). Molar stoichiometries of the identified pigments in FLAG-PSIΔC are shown in parentheses (normalized to 92 Chls and 1 Chl' in FLAG-PSI) and these values represent the means of three technical replicates; standard deviations were below 10%. ZEA – zeaxanthin, ECH – echinenone, BRC – β-carotene, a.u. – arbitrary units. **(B)** The pigment network of PSIΔC. Chlorophylls are colored green and carotenoids blue. Chl*a* 4003 (PsaK) absent in PSI is marked with a dashed square. Two Chls (CLA 1501 and 1503) and two β-carotenes (BCR 4019 and 4022) normally bound to PsaL and one β-carotene bound to PsaA (BCR 4019) are absent in PSIΔC and are colored transparent. **(C-E)** Superposition of PSIΔC and the mature model showing different co-factor occupations at specific sites. The mature PSI is coloured grey. Specifically, one β-carotene in the vicinity of PsaJ was lost and replaced by the dodecyl-β-D-maltoside molecule (LMT202). Moreover, two carotenoids were modelled as echinenones, whereas in the structure of Malavath *et al*. (2018), these densities were modelled as zeaxanthin and an isomer of canthaxanthin.

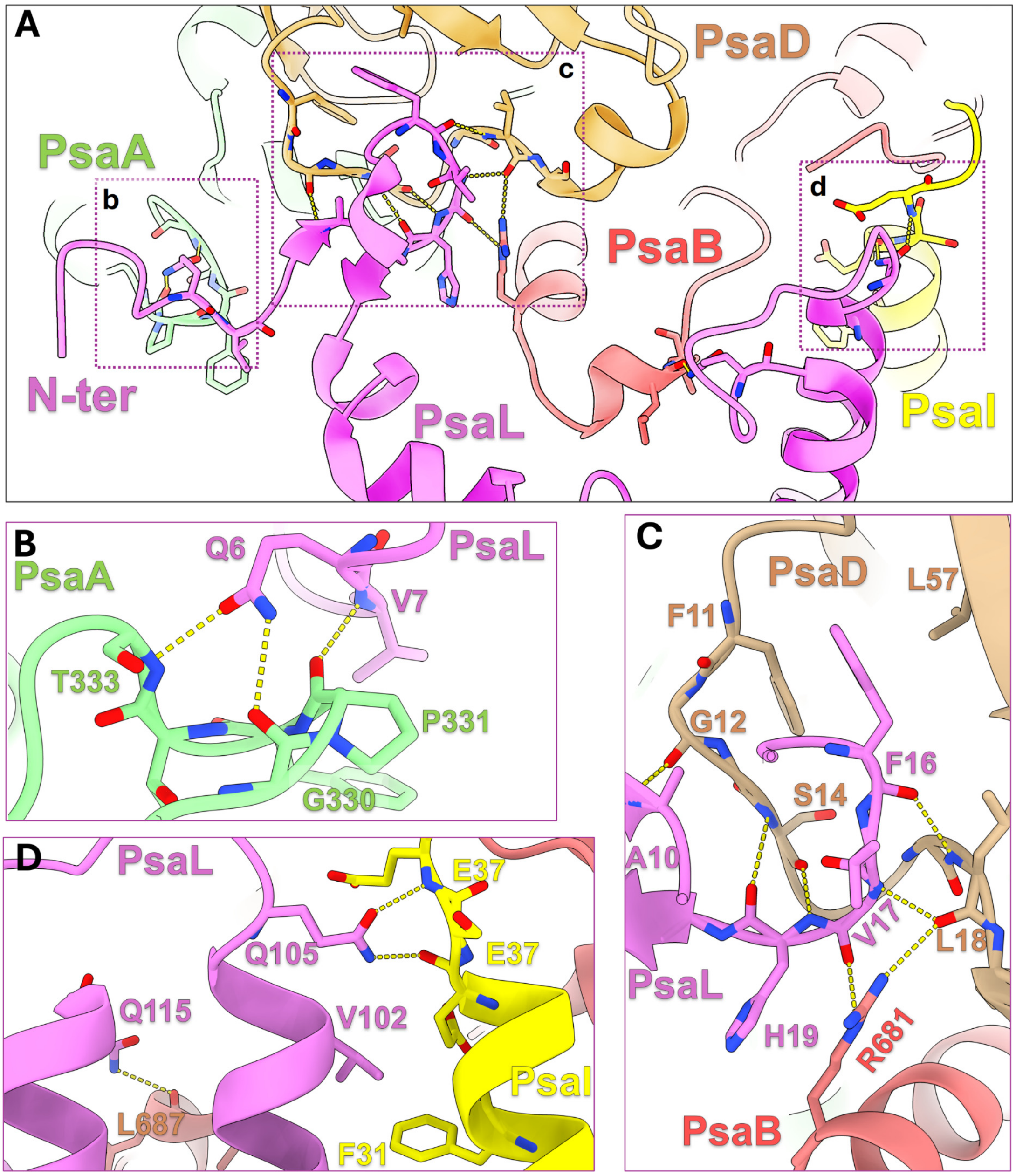

Supplemental Figure 8. Main protein–protein interactions established by PsaL with other subunits of mature PSI. (A) Cytoplasmic view of the PsaL extramembrane region forming protein-protein interaction with PsaA, PsaB, PsaD and PsaI. (B-D) close view of the protein–protein interactions marked with dotted rectangles in (A). Figures are based on previously published PSI structure (Malavath et al., 2018; PDB 5OY0).

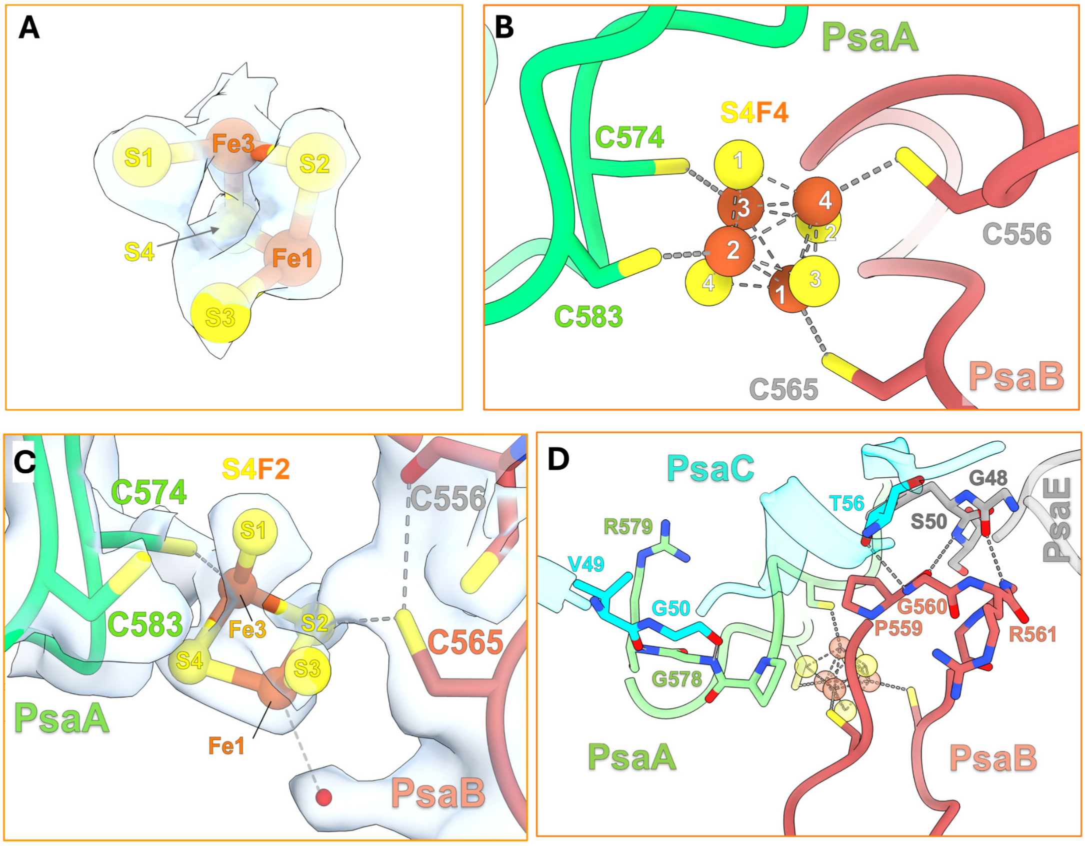

Supplemental Figure 9: Organisation of the F_X cluster in PSIΔC and the mature PSI (5OY0). (A) Model and density of the F_X cluster from the PSIΔC. (B) Coordination of the F_X cluster with cysteine residues from PsaA and PsaB in the mature PSI. (C) Coordination of the F_X cluster from the PSIΔC with cysteine residues from PsaA and PsaB. The density of the designated atoms and residues is shown. (D) Wide view of the F_X coordination site. PsaA loop, PsaB loop, PsaE, PsaC and the F_X cluster from the mature PSI are shown in light green, red, dark grey, light cyan and transparent orange-yellow, respectively. Interactions between residues are shown.

Supplemental Table 1. Mass spectrometric identification of protein bands designated in the 2D gel analysis of the FLAG–PSI preparation in Fig. 1B.

Accession number	Mass spectrometric analysis			
	Length (AA)	Size (Da)	Sequence coverage (%)	Intensity
**PsaA** P29254	751	82,950	20	38,597,000
**PsaB** P29255	731	81,292	12	16,375,000
**PsaC** P32422	81	8,828	49	3,492,300
**PsaD** P19569	141	15644	90	78,489,000
**PsaF** P29256	165	18,249	48	47,937,000
**PsaL** P37277	157	16624	11	1,035,500
**PsbE** P12975	74	8145	96	29,097,000
**PsaK1** P72712	86	8,644	20	422,860
**PsaK2** P74564	90	9,306	10	2,644,000

LC-MS/MS analysis was performed on an UltiMate 3000 UHPLC (Thermo Fisher Scientific) on-line coupled to a timsTOF Pro (Bruker Daltonics) mass spectrometer. The identifications were quantified using IBAQ quantification mode.

Supplemental Table 2. List of the most abundant 40 proteins identified by MS in the FLAG-PSI∆C preparation.

Accession numbers	Protein name	No of peptides	Intensity
P29256|PSAF_SYNY3	**Photosystem I subunit PsaF**	18	6.18E+05
P29254|PSAA_SYNY3	**Photosystem I subunit PsaA**	24	3.17E+05
P29255|PSAB_SYNY3	**Photosystem I subunit PsaB**	16	2.66E+05
P05429|PSBB_SYNY3	Photosystem II subunit PsbB (CP47)	21	1.97E+05
P72804|Y1796_SYNY3	**Thylakoid membrane protein Slr1796**	16	1.81E+05
P74227|EFTU_SYNY3	Elongation factor Tu	31	1.45E+05
P72991|FTSH3_SYNY3	ATP-dependent zinc metalloprotease FtsH3	35	1.25E+05
Q55700|FTSH2_SYNY3	ATP-dependent zinc metalloprotease FtsH2	37	1.11E+05
P73437|FTSH4_SYNY3	ATP-dependent zinc metalloprotease FtsH4	43	9.33E+04
P73690|YC51L_SYNY3	**Ycf51-like protein Sll1702**	5	8.67E+04
P74063|YCF3_SYNY3	**Photosystem I assembly protein Ycf3**	13	7.50E+04
Q55610|SECD_SYNY3	Protein translocase subunit SecD	23	6.42E+04
Q55544|APCE_SYNY3	Phycobiliprotein ApcE	48	5.99E+04
P09193|PSBC_SYNY3	Photosystem II subunit PsbC (CP43)	17	5.72E+04
P72659|PNP_SYNY3	Polyribonucleotide nucleotidyltransferase	43	5.36E+04
P72986|PSAM_SYNY3	**Photosystem I subunit PsaM**	4	4.98E+04
P36236|RL1_SYNY3	50S ribosomal protein L1 RplA	13	4.36E+04
P73308|RL5_SYNY3	50S ribosomal protein L5 RplE	16	4.32E+04
P26527|ATPB_SYNY3	ATP synthase subunit AtpD	25	4.02E+04
P74071|RS2_SYNY3	30S ribosomal protein S2 RpsB	16	3.70E+04
P73317|RL2_SYNY3	50S ribosomal protein L2 RplB	13	3.68E+04
P37277|PSAL_SYNY3	**Photosystem I subunit PsaL**	6	3.68E+04
P73306|RL6_SYNY3	50S ribosomal protein L6 RplF	14	3.63E+04
P77969|HEM2_SYNY3	Delta-aminolevulinic acid dehydratase HemB	22	3.63E+04
P73334|RPOC2_SYNY3	DNA-directed RNA polymerase subunit RpoC2	45	3.61E+04
P73304|RS5_SYNY3	30S ribosomal protein S5 RpsE	13	3.45E+04
P42352|RL9_SYNY3	50S ribosomal protein L9 RplI	12	3.41E+04
P73318|RL23_SYNY3	50S ribosomal protein L23 RplW	9	3.30E+04
P23349|RL7_SYNY3	50S ribosomal protein L7/L12 RplL	8	3.28E+04
P80505|G3P2_SYNY3	Glyceraldehyde-3-phosphate dihydrogenase 2	20	3.21E+04
P73319|RL4_SYNY3	50S ribosomal protein L4 RplD	11	3.20E+04
P73307|RS8_SYNY3	30S ribosomal protein S8 RpsH	11	3.14E+04
P73179|FTSH1_SYNY3	ATP-dependent zinc metalloprotease FtsH1	36	3.02E+04
P74564|PSAK2_SYNY3	**Photosystem I reaction center subunit PsaK2**	6	3.02E+04
P73294|RL13_SYNY3	50S ribosomal protein L13 RplM	8	3.00E+04
P73320|RL3_SYNY3	50S ribosomal protein L3 RplC	12	2.96E+04
P27179|ATPA_SYNY3	ATP synthase subunit alpha AtpA	16	2.90E+04
P73314|RS3_SYNY3	30S ribosomal protein S3 RpsC	11	2.80E+04
P09192|PSBD_SYNY3	Photosystem II subunit PsbD (D2)	5	2.77E+04
Q55709|SECA_SYNY3	Protein translocase subunit SecA	42	2.69E+04

The analysis was performed on Evosep ONE (Evosep Biosystems) connected to timsTOF SCP (Bruker Daltonics) using the Endurance column (4cm x150 µm, 1.9 µm). The identifications were quantified using IonQuant 1.10.12 with default settings for label free quantification mode. PSI=related proteins are in bold.

Supplemental Table 3. Mass spectrometric (MS) and Western blot (WB) identification of proteins in trimeric and monomeric PSI complexes of FLAG-PSI and FLAG-PSI∆C preparations separated in the CN gel shown in Supplemental Figure 4.

Protein name Accession number	Mass spectrometric analysis									
	F.PSI(3)		F.PSI(1)		F.PSI∆C(3)		F.PSI∆C(1)’		F.PSI∆C(1)	
	MS	WB	MS	WB	MS	WB	MS	WB	MS	WB
**PsaA** P29254	+	+	+	+	+	+	+	+	+	+
**PsaB** P29255	+	+	+	+	+	+	+	+	+	+
**PsaC** P32422	+	+	+	+	-	-	-	-	-	-
**PsaD** P19569	+	+	+	+	-	-	-	-	-	-
**PsbE** P12975	+	n.a.	+	n.a.	-	n.a.	-	n.a.	-	n.a.
**PsaF** P29256	+	+	+	+	+	+	+	+	+	+
**PsaI** Q55330	-	-	-	n.a.	-	n.a.	-	n.a.	-	n.a.
**PsaJ** Q55329	-	-	-	n.a.	-	n.a.	-	n.a.	-	n.a.
**PsaK1** P72712	+	n.a.	+	n.a.	+	n.a.	+	n.a.	+	n.a.
**PsaK2** P74564	+	n.a.	+	n.a.	+	n.a.	+	n.a.	+	n.a.
**PsaL** P37277	+	+	+	+	+	+	+	+	-	-
**PsaM** P72986	-	-	-	n.a.	-	n.a.	-	n.a.	-	n.a.

The analysis was performed on Evosep ONE (Evosep Biosystems) connected to timsTOF SCP (Bruker Daltonics) using the Endurance column (4cm x150 µm, 1.9 µm). n.a. means that the presence of the particular proteins was not assessed by immunodetection due to lack of appropriate antibodies. PsaI, PsaJ and PsaM were not detected by MS analysis neither, most probably due to their hydrophobic nature. Moreover, PsaI cannot be cleaved by trypsin, PsaJ theoretically gives 5 amino acid N-terminal, 9 amino acid C-terminal and 26 amino acid hydrophobic transmembrane part, all hardly detectable, and PsaM contains just a single trypsin cleavage site giving rise to hardly detectable hydrophobic 24 amino acid N-terminal and 7 amino acid C-terminal fragment.

Supplemental Table 4. Cryo-EM single particle analysis. Summary of the data collection, refinement, and validation statistics of PSIΔC (EMDB-60522) (PDB 8ZWB).

Data collection and processing	PSIΔC(1)
Magnification	165,000
Voltage (kV)	300
Electron exposure (e–/Å2)	34.41
Defocus range (μm)	-0.5 to -1.9
Pixel size (Å)	0.729
Initial particle images (no.)	9,991
Symmetry imposed	C1
Final particle images (no.)	333,941
**Map resolution (Å)**	
FSC threshold 0.143	1.83
**Refinement**	
Initial model used (PDB code)	5OY0
Map sharpening B factor (Å2)	-10.1
**Model composition**	
Non-hydrogen atoms	21,069
Protein residues	1,786
Ligands	147
Waters	355
**B factors (Å2) (min/max/mean)**	
Protein residues	19.04/118.34/43.20
Ligands	20.19/112.04/42.92
Waters	22.97/77.36/44.72
**R.m.s. deviations**	
Bond lengths (Å)	0.009
Bond angles (°)	1.023
**Validation**	
MolProbity score	1.14
Poor rotamers (%)	0.56
**Ramachandran plot**	
Favored (%)	98.42
Allowed (%)	1.58
Disallowed (%)	0.00

Supplemental Table 5. Pigment and lipids composition of each PSIΔC subunit based on the cryo-EM structure. Chls include Chl *a* (CLA) and one Chl *a'* isomer (CL0) in the reaction centre. Abbreviations: BCR - β-carotene, ECH - 5 echinenone, 5X6- zeaxanthin, LHG - 1,2-dipalmitoyl-phosphatidyl-glycerole, LMG - 1,2-distearoyl-monogalactosyl-diglycerides, and LMT - dodecyl-β-D-maltoside.

	**Chlorophylls**	**Carotenoids**			**Aliphatic molecules**		
	Chlorophyll *a*	BCR	ECH	5X6	LHG	LMG	LMT
**PsaA**	42	4	1	1	2	1	8
**PsaB**	43	5	1	-	1	1	9
**PsaF**	3	1	1	-	-	-	3
**PsaI**	0	1	-	-	-	-	2
**PsaJ**	2	1	1	-	-	-	3
**PsaK**	3	2	-	-	-	-	-
**PsaM**	-	-	1	-	-	-	1
**Total**	**93**	**14**	**5**	**1**	**3**	**2**	**26**

Supplemental Table 6. List of primers used for constructing mutants used in the study (see Supplemental Figure 1).

Primer designation	Primer name	Sequence (5’ - 3’)
F1	psaA up1 HindIII	ACAGGGAAAGCTTATACATCC
R1	psaA flag R GA	CTTTCGGGTGGACTAATTGTTGCGGCCGCTTTATCATCATC
F2	psaA F GA	ATGATGATAAAGCGGCCGCAACAATTAGTCCACCCGAAAG
R2	psaA R GA	ATTTGCAGATTATTGCTAGCCTAGCCAATGGAAAGACTGCG
F3	psaA down F GA	GCAGTCTTTCCATTGGCTAGGCTAGCAATAATCTGCAAATT
R3	psaB down1 EcoRI	GGCCGAATTCTTCTCTGATCC
F4	psaC up 800	GATACACTGGCACCAACTCCAATTCCCAGACTAGGA
R4	psaC Cm up rev	CGGTGGTATATCCAGTGATTTTTTTCTCCATTGACTATCGGC
F5	psaC up Cm fwd	GGAGCCGATAGTCAATGGAGAAAAAAATCACTGGATATACCACCG
R5	psaC Cm dw rev	ACCAGATCTATACGATCGCTTATTATCACTTATTCAGGCGTAGCAC
F6	psaC dw Cm fwd	GTGCTACGCCTGAATAAGTGATAATAAGCGATCGTATAGATCTGGT
R6	psaC dw 400	CTAATCTCCAAACGTTCTGACACCTCCTGGTTGGTCAGAC

Methods

Construction of *Synechocystis* mutant strains

In this work, we used the *Synechocystis* GT-P sub-strain (Tichý et al., 2016) as a wild-type strain. To express the N-terminally 1xFLAG-tagged PsaA (FLAG-PsaA) from the *psaA* promoter in *Synechocystis* (replacing the native *psaA* gene), a set of DNA constructs was prepared by the NEBuilder HiFI DNA assembly kit, each containing the gentamycin resistance cassette downstream of the *psaAB* locus (**Supplemental Figure 1A**). As the first step we replaced the whole *psaAB* operon to obtain strains expressing only the FLAG-PsaA lacking both native PsaA and PsaB proteins (*FLAG-psaA*/Δ*psaB* strain). The strain expressing FLAG-PsaA instead of the original PsaA (*FLAG-psaA)* was then prepared by transforming the *FLAG-psaA*/Δ*psaB* strain using the *psaB*-Gent construct and selecting for autotrophy (**Supplemental Figure 1B**). The deletion of the *psaC* gene in the *FLAG-psaA* strain was achieved by replacing most of the *psaC* gene with chloramphenicol resistance cassette (**Supplemental Figure 1B**). Segregation proceeded in low light (5 µmol photons m^{-2} s^{-1}) on plates containing glucose and particular antibiotics (Komenda et al., 2008) and was confirmed by PCR using a specific set of primers (see **Supplemental Table 6**). The correct sequence of the whole modified DNA region was confirmed by DNA sequencing in all newly constructed and fully segregated strains.

Cultivation conditions of *Synechocystis* strains

The strains were grown in 100 mL of liquid BG11 medium supplemented with 5 mM glucose using 250 mL conical flasks on a rotary shaker under 5 µmol photons m^{-2} s^{-1} at 29 °C. For protein purification, 500 mL of cell culture in a 1-L cylinder was grown under 5 µmol of photons m^{-2} s^{-1} in BG11 medium supplemented with 5 mM glucose. The cell culture was agitated with a magnetic stirrer and bubbled with air.

Absorption spectroscopy and pigment determination

Absorption spectra of the preparations were measured at room temperature using a UV-3000 spectrophotometer (Shimadzu, Japan) in the 25 mM MES/NaOH, pH 6.5, 10 mM $CaCl_2$, 10 mM $MgCl_2$, and 25% glycerol (thylakoid buffer) containing 0.05% n-dodecyl-β-D-maltoside (DDM). The spectra were normalized to the 680 nm peak of Chl. Light-induced absorption changes of the oxidized primary donor of PSI RC (P700+) in the 580-780 nm range were recorded using a locally built kinetic spectrometer described in detail previously (Bína et al. 2006). Oxidized P700 was accumulated by a 3 s pulse of blue (470 nm) light corresponding to irradiance of ~2000 µmol photons m^{-2} s^{-1} delivered by a high-power LED (M470L3, Thorlabs, USA). The sample was supplied with ~20 mM sodium ascorbate.

For routine Chl determination, pigments were extracted from cell pellets with 100% methanol and Chl concentration was determined spectroscopically (Wellburn, 1994). The amount of Chl and carotenoids per a single PSI reaction centre was determined by HPLC (Agilent 1260) using the following method: The sample was mixed with an excess of 100% methanol and the extracted pigments were separated on a reverse-phase column Zorbax Eclipse Plus C18 (4.6 x 250 mm, 5 µm, Agilent) with 32% (v/v) methanol and 14% (v/v) acetonitrile in 0.25M pyridine (solvent A) and 20% (v/v) methanol, 20% (v/v) acetone, 60% (v/v) acetonitrile as solvent B. Pigments were eluted with a linear gradient of solvent B (60–100% (v/v) in 40 min) in solvent A followed by 100% of solvent B in solvent A for 30 min at a flow rate of 0.8 ml min^{-1} at 40 °C. Chl-*a* and carotenoids were detected by a diode-array detector (Agilent 1260) at 450 nm, the obtained peaks were integrated, and the molar stoichiometries calculated from calibration curves that were prepared using authentic standards. Chl-*a* $C13^2$ epimer was detected by a fluorescence detector (Agilent 1260) set to 435 nm and 670 nm excitation and emission wavelengths, respectively.

Preparation of membranes and FLAG-specific protein purifications

Small-scale membrane fractions were prepared by breaking the cells with zirconia/silica beads using a Mini-Beadbeater (BioSpec Products, USA) according to the procedure described in (Komenda and Barber, 1995), but the cells were resuspended and broken in thylakoid buffer. Large-scale membrane preparations for the purification of proteins were carried out in thylakoid buffer containing a protease inhibitor cocktail (Roche or Sigma-Aldrich, USA) as described in (Koskela et al., 2020) with the exception that membranes were broken using Precellys Evolution (Bertin Technologies, France). Obtained membranes were solubilized with 1% DDM and FLAG-tagged proteins were isolated using the anti-FLAG M2 affinity gel (Sigma-Aldrich, USA) as described in detail in (Koskela et al., 2020).

Analysis of membrane protein complexes and their subunit composition

The analysis of membrane protein complexes using CN-PAGE in a 4% to 14% gradient polyacrylamide gel and the subsequent second dimension SDS-PAGE in a denaturing 12% to 20% gradient gel containing 7 M urea was performed as described in (Komenda et al., 2012). To increase denaturation of the complexes and identify PsaL protein, the lanes from the native gel were excised and incubated in 25 mM Tris/HCl, pH 7.5, containing 2% SDS (w/v) at 50 °C for 20 min instead of room temperature for 30 min. The separated proteins were visualized by staining with Coomassie Brilliant Blue and detected by MS. Western blots were performed as described in (Komenda et al., 2012). The primary rabbit antibodies used in the study were specific against PsaA, PsaB PsaC, PsaD and PsaL proteins (Agrisera cat. no. AS06 172, AS10 695, AS10 939, AS09 461 and AS19 4353, respectively). The antibody against PsaF was raised against residues 50-61 of the *Synechocystis* PsaF protein.

Mass spectrometry analysis and data interpretation for in-solution and in-gel samples

For analysis of the liquid PSIΔC preparation, two μl of the WT and the mutant pull-downs were diluted into the 18 μl of 50mM ammonium citrate pH 3.5 supplemented with 10% acetonitrile, 2M urea and 100mM TCEP. Subsequently, 50 ng of mucorpepsin was added to the sample and it was incubated at RT for 1hr. The desalting procedure and data-dependent mass spectrometry analysis were performed according to (Felčíková et al., 2024).

In gel digestion was performed according to Pompach et al. with minor modifications (Pompach et al., 2009). The cystamine was omitted and the in-gel reduction and alkylation was accomplished with 10mM Tris(2-carboxyethyl)phosphine and 30mM chloroacetamide at 70 °C for 10 min. After the overnight digestion, the resulting peptides were collected, the remaining gel was extracted by 5 μl of 0.5% Trifluoroacetic acid in 50% acetonitrile. The organic fraction was pulled with the previous water fraction, the peptide extracts were diluted five times by water and loaded on Evosep tips (Evosep Biosystems). The final analysis of in-gel digests was performed on Evosep ONE (Evosep Biosystems) connected to timsTOF SCP (Bruker Daltonics) using the Endurance column (4cm x150mm, 1.9 mm) and 200 method.

In both cases, data were searched using PEAKS Studio 11 (Tran et al., 2019) (Bioinformatics Solutions Inc., Waterloo, ON, Canada). The database consisted of 1,109 *Synechocystis* protein sequences (taxonomy ID: 1142, reviewed), obtained from UniProtKB (The UniProt Consortium et al., 2023) on June 14, 2024, and 243 sequences of common contaminants. Protein digestion was set to [KR|P] for native gel digests, and as unspecific for the purified preparations, with up to 2 misscleavages and allowed peptide lengths of 5-50. Protein N-term acetylation and Met oxidation were set as variable modifications, with a maximum of 2 modifications per peptide. The tolerance for precursors and fragments was set to 12 ppm and 0.05 Da, respectively. FDR thresholds were set to 1% on both the PSM and protein level. Only proteins with 2 and more unique peptides were accepted. The proteins were quantified by PEAKS as the total area of peptide features from unique supporting peptides.

Data were deposited to the ProteomeXchange Consortium via the PRIDE partner repository with the data set identifier PXD053754.

Cryo-EM single particle analysis:

Holey carbon grids for structural analysis were prepared using a PSIΔC sample with Chl concentration of 1.5 mg/ml. The grids (Cu 300 mesh 1.2/1.3, Quantifoil) were glow discharged for 60 sec with 30 mA using a GloQube (Quorum) instrument. 3 μl of the protein sample were pipetted onto the glow-discharged grids, blotted for 2.5 sec, and plunge frozen in liquid ethane using a Vitrobot Mark IV (Thermo Fisher Scientific) which was adjusted to a temperature of 4 ºC and a relative humidity of 100%. Clipped grids were imaged using a 300 kV Titan Krios G4 microscope (Thermo Fisher Scientific) that

is equipped with a Selectris Energy filter (Slit width 10 eV) and a Falcon 4i detector. Grid screening and data collection were carried out using EPU software (v3.5.1.6034). 9,991 movies were recorded at a nominal magnification of 165,000, which corresponds to a pixel size of 0.729 Å. The total dose per micrograph was 34.41 e/Å2, and a defocus range of -0.5 to -1.9 was collected. Movies were processed using the live version of cryoSPARC v4.4.0 (Punjani et al., 2017). Motion correction, CTF estimation, blob/template picking, and 2D classification were performed. *Ab initio* reconstruction was performed in cryoSPARC to get a low-resolution reconstruction. Subsequently, an intermediate-resolution reconstruction was generated using homogeneous refinement in cryoSPARC. This map and four additional junk classes were used as references in a heterogeneous refinement job using all picked particles as input. This enriched the number of good particles. The good particles were re-extracted (unbinned, 350 px box) for a subsequent non-uniform refinement job in cryoSPARC. The particle information was then converted into a Star file using csparc2star of PyEM (UCSF pyem v0.5. Zenodo). The x-y coordinates were used to re-extract particles in RELION (Scheres, 2012). Following a local angular search in 3D refinement, a 3D classification with two classes was carried out. The class with a lower particle number (333,941) but higher resolution was considered to contain better-quality particles and was used in subsequent steps. Multiple rounds of 3D refinement, followed by CTF refinement and Bayesian polishing, were conducted. Finally, a 3D reconstruction was obtained with an overall resolution of 1.83 Å (**Supplemental Figure 5**).

Model building and refinement:

Model building was carried out using Coot 9.8.9 (Emsley et al., 2010). The coordinates from the PDB entry 5OY0 were used as the starting model. The PDB was rigid-body fitted into our highest resolution map at 1.83 Å using UCSF Chimera (Pettersen et al., 2004). All deviations from the starting model, such as chain deletion, correction of rotamers, rebuilding of local sections, building of water molecules, and other improvements in model quality, were carried out using Coot. The model-to-map weight was adjusted manually according to the local quality of the map. For all ligands, restraint files were generated using the Grade2 server (http://grade.globalphasing.org). The final model was then refined using PHENIX Real-Space-Refine (Afonine et al., 2018). The refinement protocol was optimized by variations of different weights (overall weight, Ramachandran weight). Several iterations of rebuilding, refinement, and validation were carried out using Coot, Real-space-Refine, and PHENIX (Afonine et al., 2018). The final model and its corresponding maps were validated using the PDB validation server (https://validate-rcsb-1.wwpdb.org/) and were subsequently deposited into the PDB databank. Structural figures were generated using UCSF ChimeraX (Pettersen et al., 2021).

REFERENCES :

Afonine, P. V., Poon, B. K., Read, R. J., Sobolev, O. V., Terwilliger, T. C., Urzhumtsev, A., and Adams, P. D. (2018). Real-space refinement in *PHENIX* for cryo-EM and crystallography. *Acta Crystallogr D Struct Biol* **74**:531–544.

Bína D, Litvín R, Vácha F, Šiffel P (2006) New multichannel kinetic spectrophotometer-fluorimeter with pulsed measuring beam for photosynthesis research. Photosynth Res 88:351–356

Emsley, P., Lohkamp, B., Scott, W. G., and Cowtan, K. (2010). Features and development of *Coot*. *Acta Crystallogr D Biol Crystallogr* **66**:486–501.

Felčíková, K., Hovan, A., Polák, M., Loginov, D. S., Holotová, V., Díaz, C., Kožár, T., Lee, O., Varhač, R., Novák, P., et al. (2024). Design of AsLOV2 domain as a carrier of light-induced dissociable FMN photosensitizer. *Protein Science* **33**:e4921.

Komenda, J., and Barber, J. (1995). Comparison of psbO and psbH deletion mutants of Synechocystis PCC 6803 indicates that degradation of D1 protein is regulated by the QB site and dependent on protein synthesis. *Biochemistry* **34**:9625–9631.

Komenda, J., Nickelsen, J., Tichý, M., Prášil, O., Eichacker, L. A., and Nixon, P. J. (2008). The Cyanobacterial Homologue of HCF136/YCF48 Is a Component of an Early Photosystem II Assembly Complex and Is Important for Both the Efficient Assembly and Repair of Photosystem II in Synechocystis sp. PCC 6803. *Journal of Biological Chemistry* **283**:22390–22399.

Komenda, J., Knoppová, J., Kopečná, J., Sobotka, R., Halada, P., Yu, J., Nickelsen, J., Boehm, M., and Nixon, P. J. (2012). The Psb27 Assembly Factor Binds to the CP43 Complex of Photosystem II in the Cyanobacterium *Synechocystis* sp. PCC 6803. *Plant Physiology* **158**:476–486.

Koskela, M., Skotnicová, P., Kiss, É., and Sobotka, R. (2020). Purification of Protein-complexes from the Cyanobacterium Synechocystis sp. PCC 6803 Using FLAG-affinity Chromatography. *BIO-PROTOCOL* **10**.

Pettersen, E. F., Goddard, T. D., Huang, C. C., Couch, G. S., Greenblatt, D. M., Meng, E. C., and Ferrin, T. E. (2004). UCSF Chimera—A visualization system for exploratory research and analysis. *J Comput Chem* **25**:1605–1612.

Pettersen, E. F., Goddard, T. D., Huang, C. C., Meng, E. C., Couch, G. S., Croll, T. I., Morris, J. H., and Ferrin, T. E. (2021). UCSF ChimeraX: Structure visualization for researchers, educators, and developers. *Protein Sci* **30**:70–82.

Pompach, P., Man, P., Kavan, D., Hofbauerová, K., Kumar, V., Bezouška, K., Havlíček, V., and Novák, P. (2009). Modified electrophoretic and digestion conditions allow a simplified mass spectrometric evaluation of disulfide bonds. *J. Mass Spectrom.* **44**:1571–1578.

Punjani, A., Rubinstein, J. L., Fleet, D. J., and Brubaker, M. A. (2017). cryoSPARC: algorithms for rapid unsupervised cryo-EM structure determination. *Nat Methods* **14**:290–296.

Scheres, S. H. W. (2012). RELION: Implementation of a Bayesian approach to cryo-EM structure determination. *Journal of Structural Biology* **180**:519–530.

The UniProt Consortium, Bateman, A., Martin, M.-J., Orchard, S., Magrane, M., Ahmad, S., Alpi, E., Bowler-Barnett, E. H., Britto, R., Bye-A-Jee, H., et al. (2023). UniProt: the Universal Protein Knowledgebase in 2023. *Nucleic Acids Research* **51**:D523–D531.

Tichý, M., Bečková, M., Kopečná, J., Noda, J., Sobotka, R., and Komenda, J. (2016). Strain of Synechocystis PCC 6803 with Aberrant Assembly of Photosystem II Contains Tandem Duplication of a Large Chromosomal Region. *Front. Plant Sci.* **7**.

Tran, N. H., Qiao, R., Xin, L., Chen, X., Liu, C., Zhang, X., Shan, B., Ghodsi, A., and Li, M. (2019). Deep learning enables de novo peptide sequencing from data-independent-acquisition mass spectrometry. *Nat Methods* **16**:63–66.

Wellburn, A. R. (1994). The Spectral Determination of Chlorophylls a and b, as well as Total Carotenoids, Using Various Solvents with Spectrophotometers of Different Resolution. *Journal of Plant Physiology* **144**:307–313.
